# DRINKING WATER QUALITY: Better Biomarker of DBP Exposure

**DOI:** 10.1289/ehp.117-a487

**Published:** 2009-11

**Authors:** Angela Spivey

**Affiliations:** **Angela Spivey** writes from North Carolina about science, medicine, and higher education. She has written for *EHP* since 2001 and is a member of the National Association of Science Writers

When we drink chlorinated water or shower in it, we’re exposed to chemicals called disinfection by-products (DBPs), which form when organic matter in water reacts with chlorine and other treatment chemicals. Some human epidemiologic studies have found associations between exposure to high levels of DBPs and increased risk of problems such as cancer and reproductive effects. But the DBP–disease connection is difficult for scientists to fully evaluate because methods for measuring exposure have been imprecise.

Most epidemiologic studies have used water supply levels of one class of these chemicals—trihalomethanes (THMs)—as a proxy for estimating exposure. Now two studies from Weiping Zhang and colleagues published in the September 2009 issues of the *Journal of Water and Health* and of *Biomarkers* provide new evidence that urinary and blood levels of another DBP, trichloroacetic acid (TCAA), make a reliable and valid biomarker for ingestion of this and possibly other nonvolatile DBPs.

“THM levels in the water supply have been a convenient proxy for use in studies because many municipal governments already track these levels in the water supply. But indices of individual DBP exposure are needed,” says Zhang, a scientist with Alberta Health and Wellness, a division of the Canadian provincial government. Scientists have identified biomarkers for measuring THMs in exhaled breath. But THMs persist in the body for only a very short time. TCAA, on the other hand, persists in the body for several days, offering a longer window for measuring exposure, Zhang says. However, she adds, knowing only water levels of any DBP does not address how much water anyone consumes.

In Zhang’s tightly controlled study, for 15 days researchers delivered 3 L of cold tap water that contained a known amount of TCAA to each of the 46 participants. Participants were allowed to drink other beverages as desired, but when they drank water, they drank what the researchers provided. (For those who usually drank more than 3 L of water a day, the researchers provided extra TCAA-free water.) Over the course of the study the researchers collected six urine samples and four blood samples from each participant. The participants recorded their daily consumption of water and other beverages, physical activity, showering/bathing time, and use of solvent-containing products. The researchers verified water consumption by retrieving the used bottles each day and recording the volume of fluid left.

The results showed that blood levels of TCAA made a better biomarker than urinary levels, but for large epidemiologic studies, measuring urine levels is more practical because the sample is collected less invasively, says Zhang, who often conducts field work. She concludes from her results that taking urine measures over two days and averaging them is preferable to a one-day sample, as the results showed better statistical reliability with repeated measures.

Other researchers agree that the findings represent a step forward. Susan Richardson, a research chemist with the U.S. Environmental Protection Agency, says the study has many strengths, among them a large group of participants that provided more statistical power than previous studies of TCAA, careful control of TCAA ingestion, and strong correlation between TCAA levels in the ingested drinking water and levels in the participants’ urine and blood. Those elements add up to her opinion that TCAA is ready to be used as a biomarker of DBP exposure in epidemiologic studies.

Clifford Weisel, a professor of environmental and occupational health at UMDNJ–Robert Wood Johnson Medical School, suggests the TCAA biomarker would be useful in a study when combined with some measure of other routes of DBP exposure, such as showering and bathing. “These studies show that TCAA will make a potentially good biomarker of ingestion of the haloacetic acids, in particular TCAA. But measuring TCAA won’t address the issue of other exposures to DBPs such as those you get through inhalation or absorption through the skin,” he says.

TCAA isn’t a perfect biomarker. It’s not yet known whether it can indicate ingestion of the most dangerous DBPs—other classes of compounds, such as iodinated and brominated compounds, which have been found to be much more toxic than chloroacetic acids. Further study is needed to identify biomarkers for those more toxic compounds or to establish whether TCAA is a valid proxy marker for any of them, Richardson says. Weisel points out there may be sources of urinary TCAA other than drinking water; certain dry-cleaning solvents, for instance, are metabolized to TCAA once they enter the body. “It’s hard to determine if that would interfere or not, because this population had very little exposure to those solvents,” Weisel says.

Mark Nieuwenhuijsen, a research professor in environmental epidemiology at the Center for Research in Environmental Epidemiology in Barcelona, Spain, notes that choosing an exposure assessment method depends on study design. For instance, in a large population study, using THM levels in water may be preferable because collecting blood or urine samples may prove too expensive, he says. Access to subjects and timing also are important aspects to consider.

But overall, researchers call these findings a step in the right direction in an area that needs much more work. “These studies are very important because we have very few biomarkers for disinfection by-products,“ Nieuwenhuijsen says.

## Figures and Tables

**Figure:**
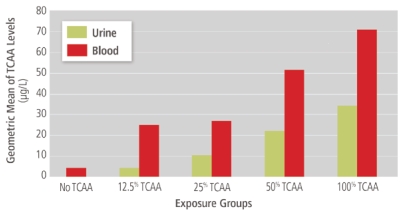
Study participants were randomly divided into 5 groups. One group served as controls and received TCAA-free bottled water. The other groups received TCAA-free water mixed with one of four concentrations (12.5%, 25%, 50%, or 100%) of city tap water containing a known concentration of TCAA. Adapted from Zhang W, et al. J Water Health 7(3):359–371 (2009), with permission from the copyright holders, IWA Publishing.

